# Supplementary material for: Geometry-Dependent Elastic Flow Dynamics in Micropillar Arrays
Source: Micromachines (Basel). 2024 Feb 13;15(2):268. doi: 10.3390/mi15020268 (PMC10893274; doi:10.3390/mi15020268)
Supplement: Supplementary file 1 [file micromachines-15-00268-s001.zip › micromachines-2825200-supplementary/ESI _pdf_and_movies/Geometry_Micromachines_8FEB2024_ESI_PROOF-2.pdf]

Electronic Supplementary Information for

Geometry-dependent Elastic Flow Dynamics in Micropillar Arrays

Oskar E. Ström, Jason P. Beech, and Jonas O. Tegenfeldt†
(Dated: February 12, 2024)

S1. DEVICE DESIGNS

The microfluidic devices were designed with the layout editing software L-Edit 16.02 (Tanner Research, Monrovia, CA, USA). The overall microfluidic device design has been reproduced from [1]. The unit cells of the dense quadratic and the hexagonal arrays are shown in Figure S1. The hexagonal array is made by shifting every other row by half a period, strictly speaking making the unit cell a stretched hexagon, although we use the shorter term throughout the paper. The sparse (quadratic) array has the same pillar diameters as the dense arrays but with a much larger pillar-pillar spacing (100 μm).

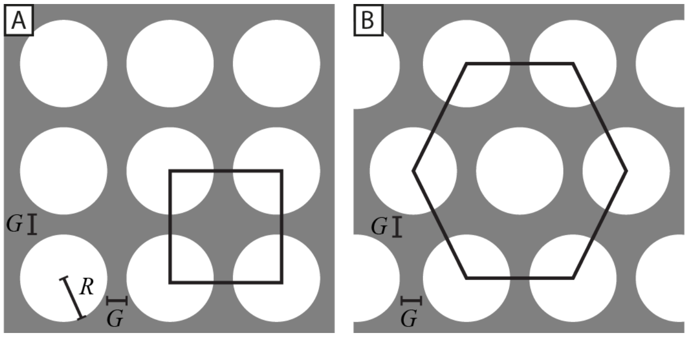

FIG. S1. Array unit cell designs of the quadratic (A) and the hexagonal (B) arrays. The pillar-pillar gap, $G \approx 4\mu m$, together with the pillar radius, $R \approx 7\mu m$, are the same for both arrays. Detailed information about the device dimensions for the different designs are given in table 1 in the main text.

The disordered array was randomized in two steps using a quadratic array as a starting point (see Figure S2 and Reference [1]). Firstly, each row of the array was shifted laterally between 0 μm and the width of a unit cell, w. Secondly, each post is moved a distance d (0 to 1.5 μm, distributed equally) in the direction θ (-180° and 180°, distributed equally). The resulting pillar gaps range from 3 μm to 6 μm.

S2. DEVICE FABRICATION

The devices were fabricated in polydimethylsiloxane (PDMS, Sylgard 184, Dow Corning, Midland, MI, USA) using standard replica molding [2]. The master mold was made in SU-8 2015 (MicroChem, Newton, MA, USA) using UV-lithography (Karl Süss MJB4, Munich, Germany) with a photomask from Delta Mask (Delta Mask, Enschede, The Netherlands). Directly following the UV-lithography, the mold was coated with an anti-sticking layer of 1H,1H,2H,2H-perfluorooctyltrichlorosilane (ABCR GmbH & Co. KG, Karlsruhe, Germany). PDMS was poured on top of the master mold to a thickness of 6 mm and cured for 1 h at 80 °C. Holes, 1.5 mm in diameter, were punched for fluidics connections. The resulting PDMS cast was thoroughly rinsed successively with isopropanol, ultra-purified water (Milli-Q® water, Merck KGaA, Darmstadt, Germany) and finally blow-dried with nitrogen gas. Coverslip glass slides (50 × 24 mm, No. 1.5H, Marienfeld, Lauda-Königshofen, Germany) were used to seal the devices. Air plasma (Zepto, Diener electronic GmbH & Co. KG, Ebhausen, Germany) was used to activate PDMS and glass surfaces. Firstly, the glass coverslips were subject to plasma for 90 s. Then, the PDMS slabs were added to the plasma oven and both surfaces were subject to an additional 10 s of plasma. The device was then finally put in an oven at 120 °C for roughly a day to render the surface hydrophobic and stored in ambient conditions according to Hung *et al.* [3] until use.

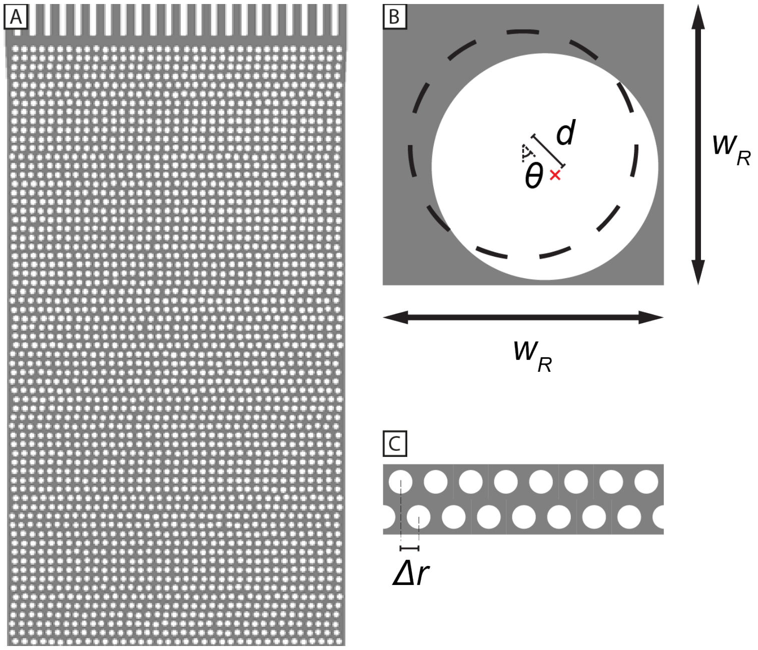

FIG. S2. Design of the disordered array. (A) Zoomed-out inlet region design. (B) Randomization on the unit cell level with w_R being the unit cell width, d, the displacement and θ the angle of displacement. (C) Randomization on the row level where the lower row has been shifted a distance Δr. The average pillar-pillar gap is $G \approx 4.5\mu m$ and the pillar radius is $R \approx 7\mu m$. Detailed information about the device dimensions are given in table 1 in the main text. Reproduced from Reference [1], DOI: 10.1039/D2LC01051H, under the terms of the CC BY 3.0 license https://creativecommons.org/licenses/by/3.0.

S3. EXPERIMENTAL SETUP

Flow in devices was generated using nitrogen overpressure which was either controlled with an MFCS-4C pressure controller (Fluigent, Paris, France) for pressures up to 1 bar or a custom-built manifold for pressures greater than 1 bar. The pressure in the custom-built setup was measured using a manometer (model 840081, Sper Scientific, Scottsdale, AZ, USA). The flow was measured by connecting the device outlet to a flow sensor (Flow unit S, FLU-S-D, Fluigent, Paris, France) with the following specifications: sensor inner diameter $150\mu m$, range $\pm 7\mu L/min$, accuracy 5% ($> 0.42\mu L/min$), and 21 nL/min ($< 0.42\mu L/min$).

The devices were imaged using an Eclipse Ti microscope (Nikon Corporation, Tokyo, Japan) and illuminated with Solis-470C High-Power LED (Thorlabs, Newton, NJ, USA) or SOLA Light Engine (6-LCR-SB, Lumencor Inc, Beaverton, OR, USA). Objectives 4× (Nikon Plan Apo λ, NA 0.20, Field of View (FoV) of 2048 μm) 10x (Nikon Plan Apo λ, NA 0.45, FoV of 819 μm), 20× (Nikon CFI Plan Apochromat λ, NA 0.75, FoV of 410 μm) and 100× (Nikon Plan Apo VC, Oil Immersion, NA 1.4, FoV of 82 μm) were used with an EMCCD camera (iXon Life 897, Andor Technology, Belfast, Northern Ireland) or a scientific CMOS camera (ORCA-Flash4.0 V3, Hamamatsu, Hamamatsu, Japan). The videos were recorded with frame rates ranging from 10 to 116 s^{-1}.

For the polarization measurement, the emitted fluorescence light was passed through an emission light splitter (OptoSplit II, Cairn Research Ltd., Faversham, UK). Two perpendicular linear polarization channels were set-up at a ± 45° angle to the long axis of the array. The emission light was thus split into two perpendicular polarizations and projected onto separate halves of the camera sensor. These halves were then overlaid using an Enhanced Correlation Coefficient (ECC) image alignment algorithm from the OpenCV package in Python. The hue, saturation and value (HSV) color model was employed to visualize the total fluorescence signal together with the polarization signal in the same image. The total fluorescence intensity of the two polarization halves is represented by the pixel value whereas the polarization emission ratio, P, represents the hue in the model. The limits of P is set to -0.2 and 0.2, and translated to 0 (red) and 1/3 (green) in the HSV model. The saturation level is set to 1 for all pixels, not containing any information about the fluorescent micrographs.

The ambient temperature in the close proximity of the device was measured to 22 ± 1.4 °C.

S4. SAMPLE PREPARATION

The DNA solution, lambda phage DNA (λ-DNA, 48.5 kbp, New England Bio-labs, Ipswich, MA, USA) was diluted to 400 μg/mL in a filtered (200 nm pore) buffer of 5× Tris EDTA (50 mM Tris-Hcl and 5 mM EDTA, pH 8) and stained 50:1 with the bisintercalating dye YOYO-1 iodide (491ex/509em, Life Technologies, Carlsbad, CA, USA) for 2 h at 50°C. The solution was stored in the fridge for a few days until use. The λ-DNA sample was heated to 65 °C for 10 min and then rapidly cooled in an ice bath to remove concatemers.

S5. IMAGE PROCESSING AND ANALYSIS

The data is processed in various ways to demonstrate the spatial and temporal variation of the DNA as it flows through the pillar arrays. Snapshots as well as kymographs are presented in the main text. In an attempt to create fingerprints for each type of flow pattern, we made scatter plots of the mean value versus the standard deviation of individual pixels for the different array types (see Figure S3). However, the main quantitative analysis is performed using Fourier analysis of the spatial variations as well as the temporal evolution of the patterns that we observe (see Section S5 A).

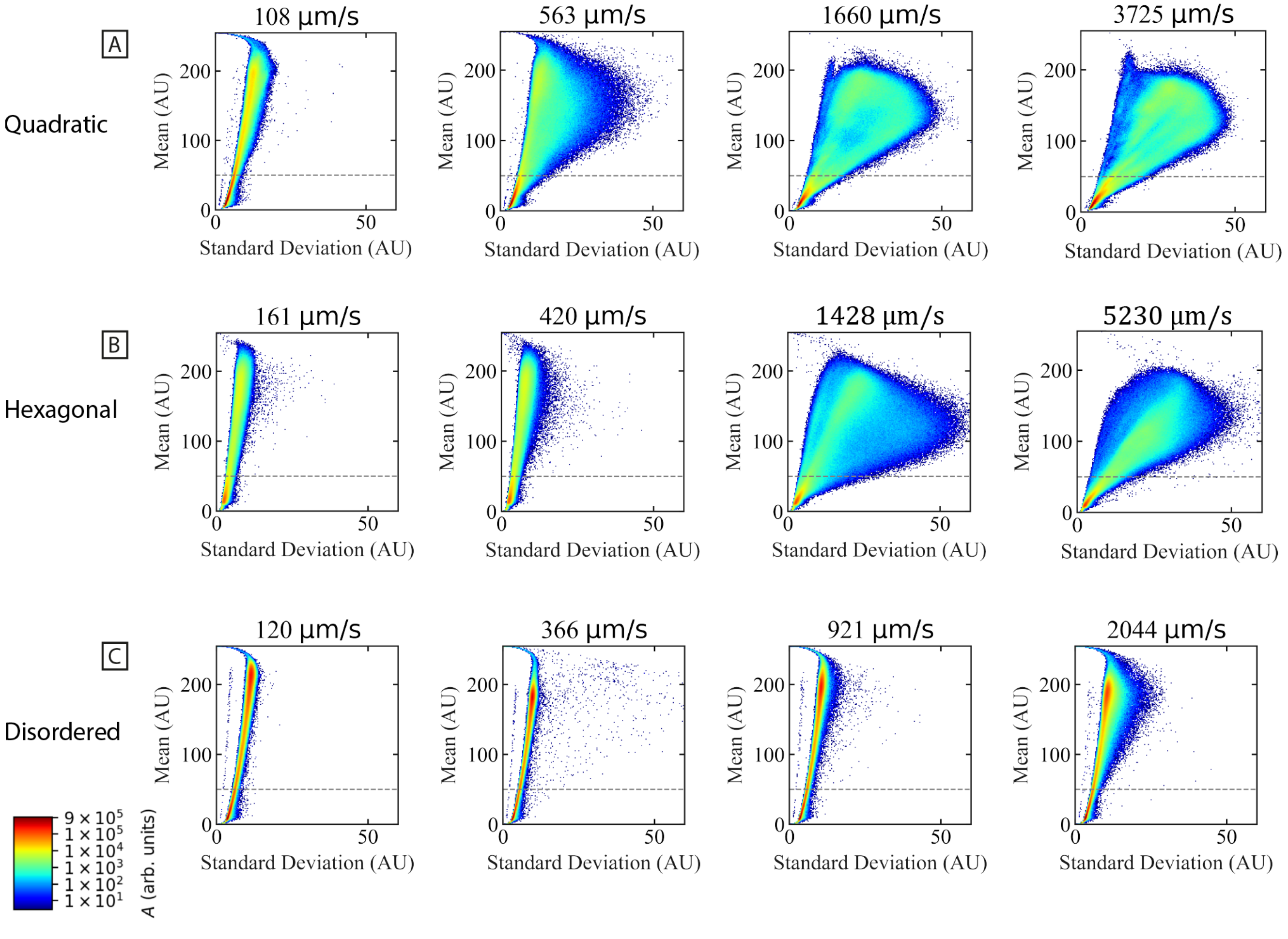

FIG. S3. Density heat maps of the mean intensity plotted against the standard deviation of the intensity for each pixel in each frame in the low resolution (4×) videographs for the three dense pillar-array geometries studied (A-C). Image size is 2048 × 488 pixels with a total of 500 frames at a constant exposure time of 30 ms but with frame rates that are adjusted with the flow rate. The frame rates were adjusted to higher values for higher flow rates, thus shortening the total acquisition time. See Table S1.

TABLE S1. Applied pressures and frame rates for the three different geometries for the data shown in Figure S3. The pressures are adjusted according to the frequency of the waves. At higher pressures, when the wave frequencies are higher, the frame rate is increased.

**Quadratic**		**Hexagonal**		**Disordered**	
P (mbar)	Frame rate (fps)	P (mbar)	Frame rate (fps)	P (mbar)	frame rate (fps)
50	1.86	59	1.86	25	1.89
200	12.54	118	5.16	50	5.27
500	20.6	471	20.63	100	20.8
1000	33.33	2000	33.33	200	20.63

A. Spatial and Temporal Frequency Analysis

Two-dimensional, spatial, fast Fourier transform (FFT) from the python package NumPy (numpy.fft.fft2) was applied to every frame of the background-subtracted low-magnification (4× fluorescent video data. The zero-frequency terms were shifted to the center of the image using the function numpy.fft.fftshift. To emphasize the features in the Fourier spectrum that can be traced to the waves, each Fourier spectrum is normalized by subtraction by the Fourier spectrum of data for the case of the smallest applied pressure (see Figure 3 in the main text).

Temporal FFT was performed by analyzing the time series of intensity within a circle with a diameter corresponding to approximately the width (163 μm) of a typical wave as seen in the quadratic device (see Figure S4). The results are averaged row by row and used to quantify the distance along the device required for the waves to form. The amplitude at the inverse relaxation time ($\tau^{-1} = 0.699$ Hz) is shown along the device channel (see Figure 4 in the main text). Figure S5 shows that it is sufficient to consider rows of circles that are positioned side by side and that do not overlap. Averaging instead the Fourier transforms of 5 × 41 circular regions across the entire field of view (see Figure S4A), the Fourier amplitude is plotted as a function of frequency (see Fig. 5 in the main text). The variation of the amplitude among individual circles is depicted in Figure S6. Here the power law exponent is derived from power law fits of the type $y = m \cdot x^k$, where m and k are fit parameters.

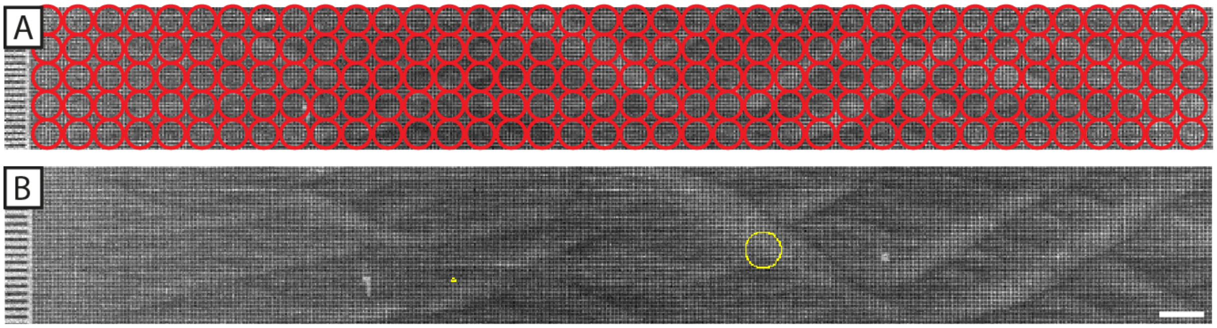

FIG. S4. Layout of the computational analysis used to characterize fluctuations. (A) The entire Field Of View is divided into a square grid consisting of circles of a radius of 81.5 μm. The mean intensity fluctuations over time are analyzed for each circle in the grid (see Figures 4 and 5 in the main text for results). (B) A single circle to show the relationship of the diameter to the size of the waves. Note that the actual plotted data is based on the average of the Fourier transforms for the intensity in each circle in each column of circles. The scale bar is 200 μm.

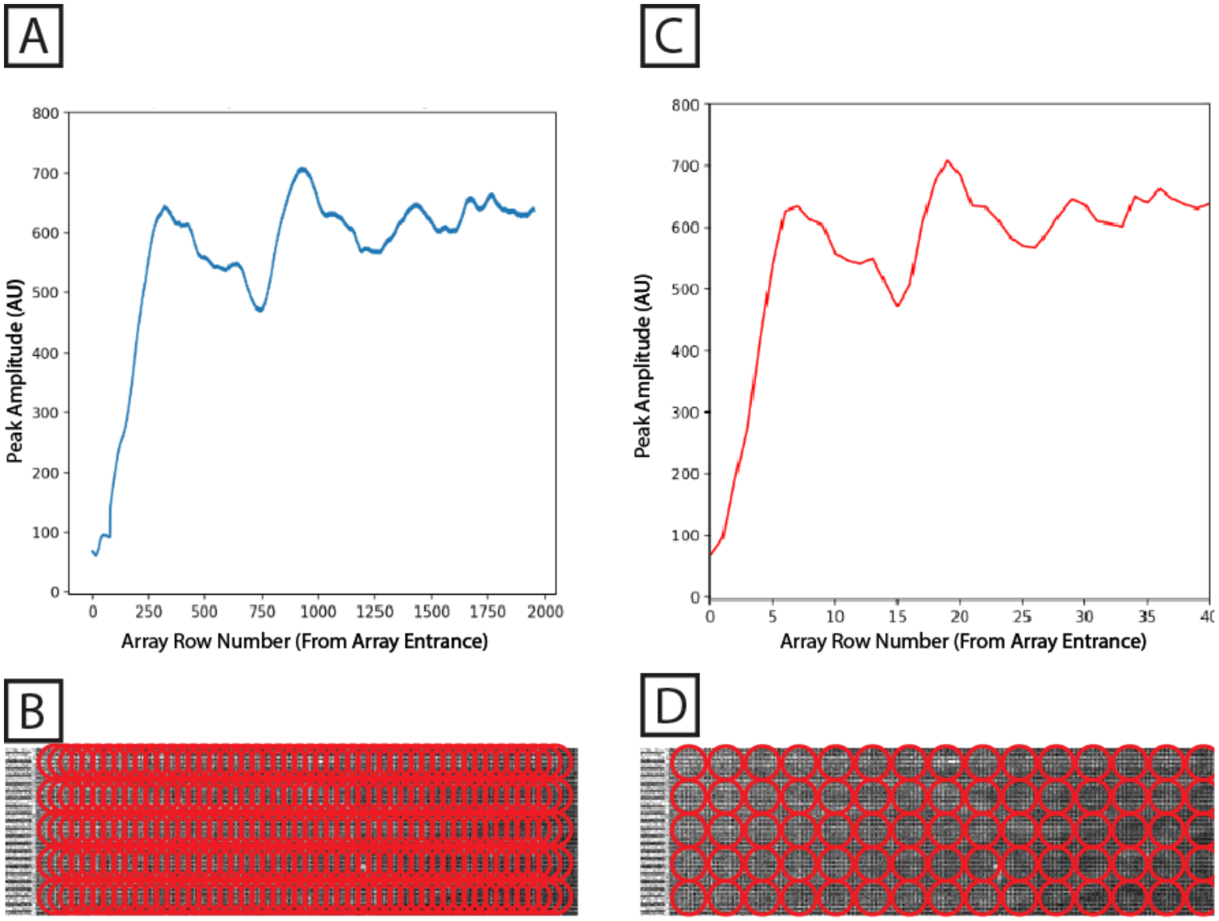

FIG. S5. Increasing the density of the measurement-region of interest (ROI) does not improve the data resolution significantly for $u \approx 3700$ μm/s in the quadratic array. In other words, it is sufficient to have a sparse sampling of the data. The plots show the amplitudes for the inverse relaxation time ($\tau^{-1} = 0.699$ Hz) along the device channel, similar to what is shown in Figure 4 in the main text but with a linear scale for the amplitude and without the averaging over a range of frequencies. Row number refers to rows of circles defining the regions where the Fourier transform takes place. (A, B) Dense shift in region sampling. Each measurement row of circles is shifted 1 pixel or 3.25 μm. (C, D) Sparse shift in region sampling. Each measurement row is shifted 50 pixels or 163 μm corresponding to one circular region diameter.

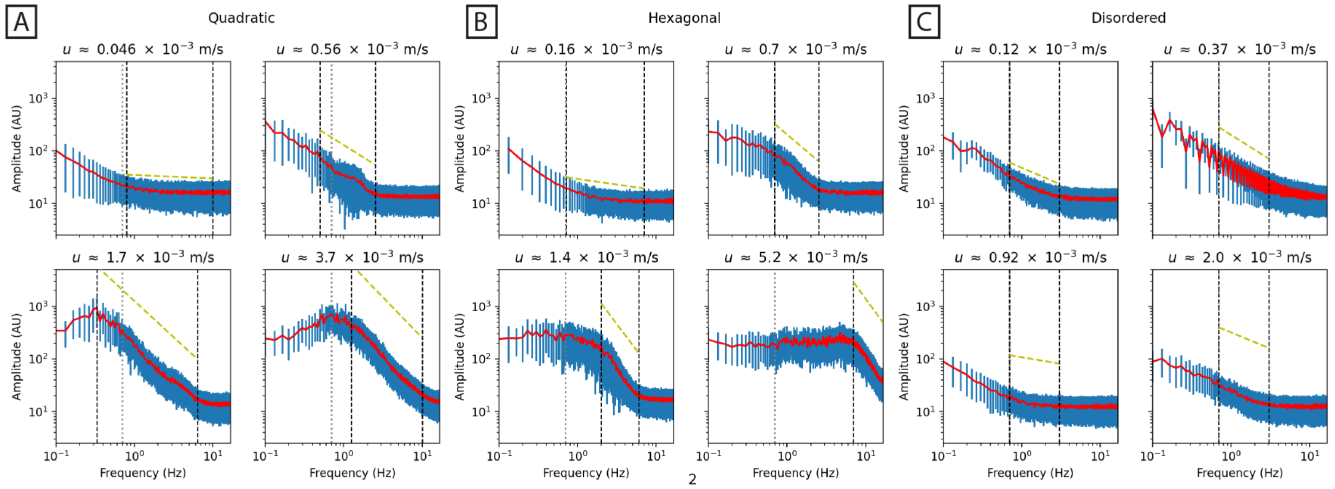

FIG. S6. Temporal Fourier spectra for the quadratic, hexagonal and disordered arrays for different flow velocities. The red line corresponds to the mean intensity for a given frequency in the 5 × 41 circular regions presented in Figure S4A. The blue lines corresponds to the standard deviation of the frequency across all circular regions. The dashed yellow lines represent the closest fits to power laws in the frequency range with boundaries marked by the vertical black dashed lines. The exponents correspond to k in the power law $y = m \cdot x^k$ and are given in the order from lowest to highest flow velocities. The inverse relaxation time is marked as a vertical gray dotted line. (A) Quadratic array for the flow velocities 46 μm/s, 560 μm/s, 1700 μm/s, and 3700 μm/s. The exponents are -0.06, -0.93, -1.38, -1.54. (B) Hexagonal array for the flow velocities 160 μm/s, 720 μm/s, 1400 μm/s, and 5200 μm/s. The exponents are -0.22, -1.27, -1.98, -2.04. (C) Disordered array for the flow velocities 120 μm/s, 370 μm/s, 920 μm/s, and 2000 μm/s. The exponents are -0.64, -0.94, -0.25, -0.62.

S6. SUPPLEMENTAL MOVIES

Supplemental Movie S1. Low magnification (2×) fluorescence videographs of the λ-DNA solution flowing across the quadratic (3700 μm/s, $De = 470$), hexagonal (5200 μm/s, $De = 680$), and disordered arrays (6700 μm/s, $De = 830$) at high flow rates.

Supplemental Movie S2. High magnification (100×) fluorescence polarization videograph of the λ-DNA solution flowing around a single pillar in the sparse array at high flow rate (6100 μm/s, $De = 87$).

Supplemental Movie S3. Intermediate magnification (20×) fluorescence videograph of the λ-DNA solution flowing across the disordered array at high flow rate (36 mm/s, $De = 4500$).

Supplemental Movie S4. Low magnification (10×) fluorescence videographs of DNA blobs being continuously generated and shed at high flow rate (11 mm/s, $De = 160$) in the sparse array.

[1] O. E. Ström, J. P. Beech, and J. O. Tegenfeldt, Short and long-range cyclic patterns in flows of DNA solutions in microfluidic obstacle arrays, Lab on a Chip **23**, 1779 (2023).
[2] Y. Xia and G. M. Whitesides, Soft lithography, Angewandte Chemie International Edition **37**, 550 (1998).
[3] L.-H. Hung and A. P. Lee, Optimization of droplet generation by controlling pdms surface hydrophobicity, in *ASME International Mechanical Engineering Congress and Exposition*, Vol. 47039 (2004) pp. 47–48.
